# Identification of *Macadamia integrifolia* Leaf Blight Disease Caused by *Pestalotiopsis colombiensis* in China

**DOI:** 10.3390/jof11090613

**Published:** 2025-08-22

**Authors:** Huizhi Yu, Youyan Lei, Ling Ma, Xiahong He, Wenhao Dai, Jie Chen, Xin Hao

**Affiliations:** 1 Yunnan Provincial Key Laboratory for Conservation and Utilization of In-forest Resource, Southwest Forestry University, Kunming 650224, China; 18332727833@163.com (H.Y.); hxh@swfu.edu.cn (X.H.); 2 Key Laboratory of Forest Disaster Warning and Control of Yunnan Province, Southwest Forestry University, Kunming 650224, China; 19887991614@139.com; 3 School of Forestry, Northeast Forestry University, Harbin 150040, China; maling63@163.com; 4 Key Laboratory of National Forestry and Grassland Administration on Forest and Grassland Pest Monitoring and Warning, Center for Biological Disaster Prevention and Control, National Forestry and Grassland Administration, Shenyang 110034, China; dwh311026@163.com

**Keywords:** *Macadamia integrifolia*, *Pestalotiopsis colombiensis*, leaf blight disease

## Abstract

*Macadamia integrifolia*, a tropical and subtropical fruit tree with significant economic and nutritional value, faces serious fungal disease problems during cultivation that severely affect yield and quality. In November 2024, leaf blight symptoms of *M. integrifolia* were observed in Menglian, Pu’er, Yunnan, China, with a disease incidence of 23% in the field. Initial symptoms included small spots that enlarged into circular to irregular lesions with red-brown centers and brown to black margins. Finally, the leaves turned yellow and became scorched, eventually leading to massive leaf shedding. Infected leaf samples were collected, and fungal strains were isolated, purified, and inoculated via spore suspension, followed by re-isolation. The strains were conclusively identified as *Pestalotiopsis colombiensis* (SWFUCB2, SWFUCB1) through an integrated approach combining DNA extraction, polymerase chain reaction (PCR), sequencing, phylogenetic reconstruction, and morphological characterization. This is the first report of *P. colombiensis* causing *M. integrifolia* leaf blight disease in China, filling a gap in research on this disease. This study provided important information for epidemiological research on this disease and the development of comprehensive leaf blight disease control strategies.

## 1. Introduction

*Macadamia integrifolia*, a member of the Proteaceae family, is a tall-canopy evergreen tree characterized by smooth leaves with serrated margins. Native to the coastal subtropical rainforests of Australia, this species is now primarily distributed across Oceania, Southeast Asia, and China. Its kernels are nutrient-rich, containing high levels of unsaturated fatty acids, protein, minerals, and vitamins. These kernels also exhibit antioxidant, lipid-lowering, and anti-inflammatory properties, contributing to their significant economic value and earning them the title of the “King of nuts”. Leaf diseases impair photosynthesis, thereby reducing the synthesis and accumulation of photosynthetic products and ultimately affecting both fruit yield and quality [1]. With the expansion of cultivation areas, various fungal diseases have increasingly emerged in crops. Currently, known diseases of *M. integrifolia* include canker disease [2], leaf spot disease [3], dieback disease [4], anthracnose [5], and black rot and flower blights [6], all of which result in substantial economic losses. Therefore, timely detection, accurate identification, and effective management of these diseases are essential. Such research enhances macadamia yield and quality and also plays a critical role in the sustainable development of the macadamia industry.

In 1949, Steyaert classified the genus *Pestalotia* spp. into three distinct genera—*Pestalotia* spp., *Pestalotiopsis* spp., and *Truncatella* spp.—based on the morphology of their conidia [7]. Species within *Pestalotia* spp. are necrotrophic plant pathogens capable of infecting a wide range of host plants, causing diseases such as leaf spot, gray mold, and leaf blight. Studies have demonstrated that fungi in this genus can produce various enzymes and toxins that degrade plant cell walls, facilitate nutrient acquisition, and induce plant tissue necrosis [8]. *Pestalotiopsis colombiensis* is a member of the broader *Pestalotia* complex and belongs to the genus *Pestalotiopsis*. *Pestalotiopsis* spp. are typical phytopathogens with a broad host range, responsible for numerous plant diseases including canker [9], leaf blotch [10], trunk disease [11], rot disease [12], dieback [13], and various post-harvest diseases. Morphologically, *Pestalotiopsis* spp. produce fusiform conidia with four septa (five cells), where the basal and terminal cells are hyaline, and the median cells are brown. The apical cell is cylindrical to subcylindrical, hyaline, thin-walled, and smooth, with tubular apical appendages [14]. These conidia are formed through budding, representing the asexual reproductive stage of the fungus. Due to its diverse dispersal mechanisms, *Pestalotiopsis* is widely distributed across various ecological environments.

In recent years, *Pestalotiopsis* and *Neopestalotiopsis* have emerged as significant pathogens of *M. integrifolia*, causing leaf and flower diseases across all major production regions worldwide. Initial symptoms are irregular light-brown leaf spots that expand and coalesce [15,16]; flower infections appear as necrotic blight and rachis dieback, collectively termed “dry flower disease” [17]. These pathogens have been recorded in diverse regions: *N. clavispora* caused severe leaf spot epidemics in Brazil [16] and both leaf spots and flower blight in China [15]. In Australia, five novel *Neopestalotiopsis* species—*N. drenthii*, *N. maddoxii*, *N. olumideae*, *N. vheenae* and *N. zakeelii*—were isolated from diseased macadamia inflorescences [6], alongside the previously described *P. macadamiae* and *N. macadamiae* as primary agents of dry flower disease [17]. Collectively, *Pestalotiopsis* and *Neopestalotiopsis* form a widespread, genetically diverse pathogen complex affecting both vegetative and reproductive tissues of macadamia, with major implications for global disease management.

Yunnan is the main *Macadamia*-producing region in China. Yunnan’s sub-tropical plateau monsoon climate aligns well with the plant’s environmental requirements, creating a natural haven for macadamia nut growth. *Macadamia* trees grow well in humid, relatively low-fertility soil and areas with annual rainfall exceeding 1000 mm, summer temperatures under 39 °C, and frost-free winters [18]. In November 2024, leaf blight symptoms of *M. integrifolia* were observed in Menglian, Pu’er, Yunnan, China (22°19′43.6″ N, 99°35′30.2″ E), with a disease incidence of 23% in the field. This is the first report of *P. colombiensis* causing leaf blight on *M. integrifolia* in China. It represents a significant advancement in understanding the etiology of this economically important disease. The findings provide a critical foundation for developing targeted and sustainable disease management strategies, which are essential for safeguarding Yunnan’s macadamia industry and ensuring its long-term productivity and economic viability.

## 2. Materials and Methods

### 2.1. Sample Collection and Isolation

In November 2024, diseased leaves of *M. integrifolia* (Disi 1) were collected from Menglian, Pu’er, Yunnan, China (22°19′43.6″ N, 99°35′30.2″ E). A five-point sampling method was employed during the field survey, covering a total of 50 trees. From each tree, 20 leaves were collected from four cardinal directions—east, south, west, and north—to investigate the occurrence of foliar diseases. Subsequently, 20 diseased leaves were randomly selected and examined under a microscope (Olympus CX33, Nagano, Japan). To obtain a pure fungal isolate, diseased leaves exhibiting typical spot symptoms were surface-sterilized with 75% ethanol for 10 s followed by 1% sodium hypochlorite (NaClO) for 40 s. The leaves were then rinsed three times with sterile distilled water and air-dried on sterilized filter paper. Using insect needles under microscopic guidance, individual conidia were isolated and transferred to potato dextrose agar (PDA; 200 g potato, 20 g glucose, and 17 g agar per liter) (Beijing Land Bridge Technology Co., Ltd., Beijing, China). Once single colonies developed, they were subcultured onto fresh PDA slants for preservation and incubated at 25 °C [19]. Pure cultures were obtained through single-spore isolation, resulting in six fungal strains. The representative specimens were deposited at Southwest Forestry University.

### 2.2. Morphological Identification

Morphological features were observed under an optical light microscope (Olympus CX33). The length, width, and morphological characteristics of conidia and conidiophore were measured (*n* = 50).

### 2.3. DNA Extraction, Polymerase Chain Reaction and Sequencing

Mycelial plugs (0.5 cm in diameter) were cultured on potato dextrose agar (PDA) for 5 days. Three plugs were used to inoculate 100 mL of liquid potato dextrose (PD) medium (200 g/L potato and 20 g/L dextrose). The cultures were incubated at 25 °C in the dark with 120 rpm for 8 days. Then, 100 mg of mycelium was collected, and total DNA was extracted using a Plant Genomic DNA Kit (Tiangen Biotech, DP305, Beijing, China).

The integrity of the extracted DNA was assessed using 0.8% agarose gel electrophoresis. The internal transcribed spacer (*ITS*) regions, large subunit ribosomal RNA gene (*LSU*), and translation elongation factor 1-alpha gene (*TEF1-α*) were amplified via PCR, respectively (Table 1). The reaction mixture for PCR contained 12.5 μL of 2 × Taq PCR Mix (Tiangen Biotech, T201), 1 μL of DNA sample, 1 μL each of the forward and reverse primers, and double-distilled water at a volume up to 25 μL. The PCR cycling conditions were as follows: 94 °C for 3 min; 32 cycles of 94 °C for 30 s, 58 °C for 30 s, and 72 °C for 2 min; and a final extension at 72 °C for 10 min. All primers were synthesized by Sangon Biotech Co., Ltd. (Shanghai, China). The amplified products were analyzed by means of agarose gel electrophoresis and sequenced using the Sanger method by Sangon Biotech Co., Ltd. (Shanghai, China).

### 2.4. DNA Sequence Analysis

The genetic sequences were analyzed and compared to existing sequences in GenBank using BLAST(2.17.0). These sequences, along with reference sequences retrieved from GenBank, were subsequently manually edited and aligned using MEGA (11) [25]. Phylogenetic relationships were inferred through the maximum likelihood method based on a heuristic search strategy. Bootstrap support values were estimated with 1000 replicates to assess the reliability of tree branches [26]. Sequence identity (%) was calculated using the MegAlign program (17.6) (DNASTAR Inc., Madison, WI, USA).

### 2.5. Pathogenicity Test

Twenty attached leaves (Disi 1) were inoculated via needle puncture with the CB1 conidial suspension (1 × 10^6^ spores/mL), and another twenty control leaves were inoculated with sterile water at 26 °C and 70% relative humidity. After 15 days, the onset of disease was observed in both treated and control groups. When symptoms developed, the pathogen was reisolated from the infected tissues and compared to the original inoculated strain based on morphological and molecular characteristics.

## 3. Results

### 3.1. Sample Collection

**Figure 1:**
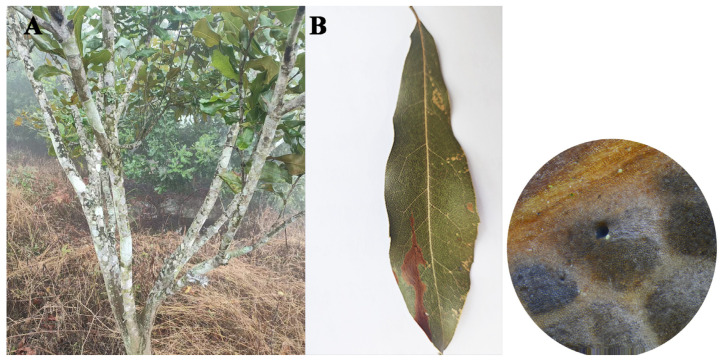
*M. integrifolia* leaf blight field symptoms. (**A**) *M. integrifolia* leaf blight field symptoms; (**B**) symptoms of *M. integrifolia* leaf blight.

In November 2024, a total of 80 leaf blight cases were observed in *Macadamia integrifolia*, with diseased leaf incidence of 23% in the field (Figure 1A). Initial symptoms appeared as small spots that expanded into circular to irregular lesions, characterized by red-brown centers and brown to black margins. As the disease progressed, leaves turned yellow and exhibited a scorched appearance, ultimately resulting in extensive defoliation (Figure 1B).

### 3.2. Morphological Data

**Figure 2:**
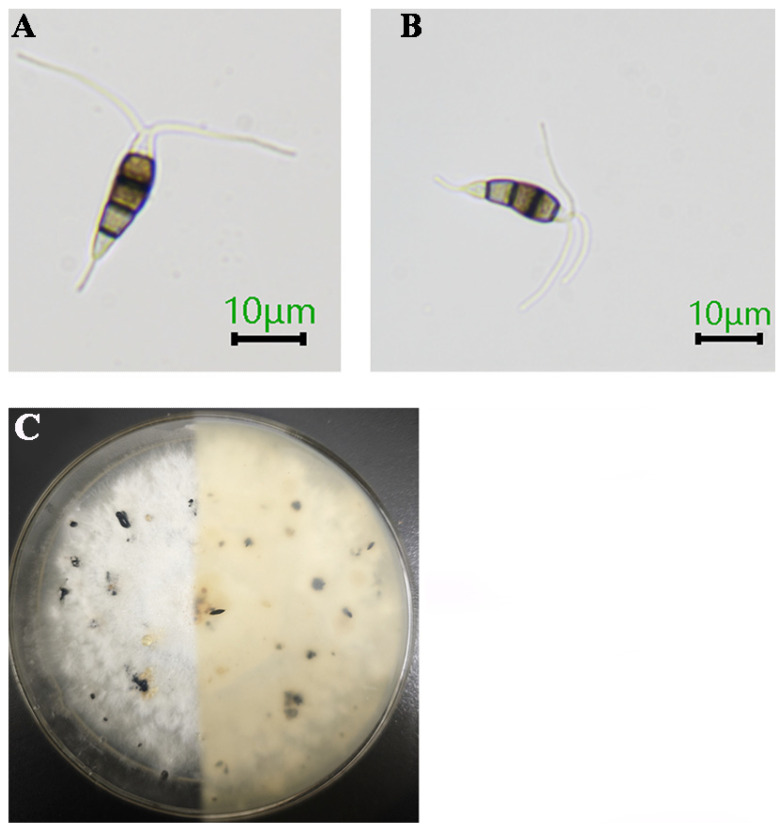
Symptoms of *Pestalotiopsis colombiensis*. (**A**) Conidia with two appendages; (**B**) conidia with three appendages; (**C**) colony on PDA after 25 days; scale = 10 μm.

Six strains were obtained through single-spore isolation. The CB1 colonies exhibited a thick and villous morphology, with white aerial surfaces and yellowish undersides. Acervuli were spherical or club-shaped, scattered or semi-emergent to emergent, dark brown to black in color, and reached diameters of up to 650 μm on PDA medium after 15 days (Figure 2C). Conidia were fusiform and four-septate, with dimensions measuring 18.79–28.25 × 5.54–9.00 μm. The basal and terminal cells were hyaline, while the three median cells were brown, with a combined length of 13.00–17.05 μm. Individual measurements for each median cell were as follows: second cell: 4.44–6.12 μm; third cell: 4.14–5.59 μm; fourth cell: 4.14–6.00 μm. The apical cell was cylindrical to subcylindrical, hyaline, and thin-walled, bearing two to three unbranched, filiform, tubular appendages 12.74–28.89 μm in length, extending from the apical crest. A single, central, tubular basal appendage 3.26–5.00 μm in length was observed (*n* = 50). These morphological features were consistent with those for *P. colombiensis* (Figure 2A,B).

### 3.3. Phylogenetic Analyses

The internal transcribed spacer (*ITS*), translation elongation factor 1-alpha (*TEF1-α*), and large subunit ribosomal RNA gene (*LSU*) partial genes were amplified. BLAST analysis of the sequence showed a homology of 99.13% (573/578), 100.00% (868/868), and 100.00% (475/475) with *P. colombiensis* (KU715149, NG069213 and KM199488).

**Figure 3:**
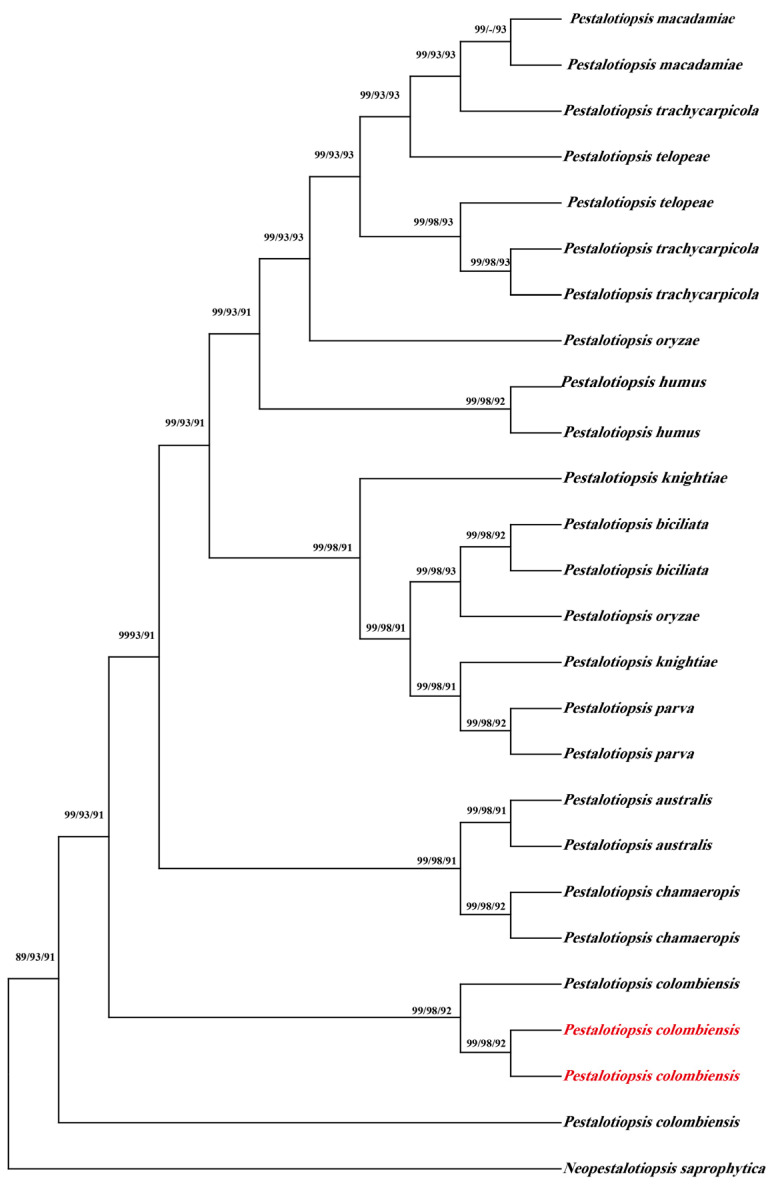
Phylogram inferred from neighbor-joining analysis of combined *ITS*, *LSU* and *TEF1-a* sequence data. The values above the branches indicate bootstrap values. The percentage of replicate trees in which the associated taxa clustered together in the bootstrap test (1000 replicates) is indicated next to the branches. Numbers above branches show bootstrap support values. Newly obtained sequences in this study are depicted in red.

All sequences were deposited in GenBank (*ITS*: PQ895603 (CB1), PQ895604 (CB2); *LSU*: PQ895622 (CB1), PQ895623 (CB2); *TEFl-α*: PQ997934 (CB1), PQ997935 (CB2)). Phylogenetic analysis using the neighbor-joining analysis with concatenated sequences was performed using MEGA 10. The sequences were aligned with 76 sequences from 12 taxa retrieved from GenBank (Table 2). The resulting phylogenetic tree shows strong bootstrap support for *P. colombiensis* (Figure 3).

### 3.4. Pathogenicity Test Using M. Integrifolia Leaves

**Figure 4:**
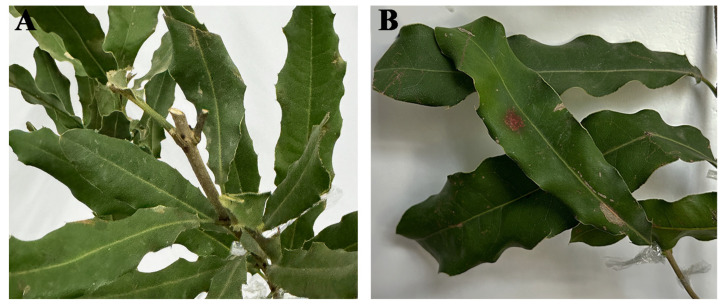
Symptoms of *P. colombiensis* and symptoms of re-inoculated leaves after 15 days and sterile water processing. (**A**) Control; (**B**) re-inoculated.

To confirm the pathogenicity of the isolate, twenty attached leaves were inoculated with a conidial suspension (1 × 10^6^ conidia/mL), while the control group was inoculated with ddH_2_O (Figure 4A). After 15 days, the inoculated leaves displayed typical blight symptoms resembling those seen under natural field conditions, whereas the control leaves remained asymptomatic. The strain CB2 was reisolated from the re-inoculated leaves, and the morphological and molecular characteristics were consistent with those of *P. colombiensis* CB1 (Figure 4B).

## 4. Discussion

*Pestalotiopsis* spp. is known to cause a wide range of plant diseases, including canker disease, leaf blotch disease, trunk disease, rot disease, dieback disease, and various post-harvest disorders. Although *Pestalotiopsis* species are recognized as pathogens of numerous host plants, there have been no prior reports regarding the pathogenicity of *P. colombiensis*. *Macadamia integrifolia* is among the susceptible hosts, as *Pestalotiopsis* spp. have been implicated in several diseases affecting this crop. For instance, *P. macadamiae* causes dry flower disease in Australia [17], and *Pestalotiopsis*-induced macadamia flower blight has also been documented. Additionally, *P. clavispora* is associated with husk rot in macadamia [27]. However, until now, *Pestalotiopsis* spp. has not been reported to cause leaf blight on *M. integrifolia*, and *P. colombiensis* has not previously been linked to any plant disease. Therefore, this study provides the first evidence and foundational insights into the pathogenic potential of *P. colombiensis*.

A spectrum of foliar fungal disorders have been documented on macadamia leaves, such as yellow halo leaf blight [3], brown leaf blight [28], leaf anthracnose [16], black leaf blight [29], and leaf spot [30], as detailed in several recent reports. This study provides important insights into the distribution and etiology of macadamia leaf blight in China. The initial symptoms manifested as small spots that expanded into circular or irregular lesions, characterized by red-brown centers and brown to black margins. To date, leaf spot disease caused by *Neofusicoccum parvum* has been reported on *Macadamia integrifolia* in Lincang, China. These lesions, measuring 3 to 5 mm in diameter, appeared as small, round brown spots with yellowish edges [31], represented another foliar disease affecting macadamia leaves. Additionally, leaf blight caused by *P. trachicarpicola* has been documented on *Panax notoginseng* in China. Its early symptoms included water-soaked discoloration, which later developed into dry yellow areas accompanied by slight leaf shrinkage [32]. As the disease progressed, leaf shrinkage and discoloration intensified, ultimately leading to leaf necrosis and defoliation. Based on the distinct symptomatology, this study differentiates the current disease from previously reported cases and identifies it as *M. integrifolia* leaf blight. Morphological and molecular analyses both clearly indicate that the causal agent belongs to the species *P. colombiensis*. This represents the first confirmed report of *P. colombiensis* causing leaf blight on *M. integrifolia* in China. Previous records of *P. colombiensis* on *M. integrifolia* lacked sufficient documentation, but this study now confirms its pathogenicity with confidence. Therefore, *M. integrifolia* should be formally recognized as a host species of *P. colombiensis*. These findings contribute valuable data for future epidemiological investigations and the development of targeted management strategies for the macadamia industry.

Currently, *Pestalotiopsis* has attracted significant research attention due to its ability to secrete a wide range of biologically active metabolites [33]. In addition to beneficial compounds, some species within this genus are known to produce phytotoxic secondary metabolites [34]. *P. longiseta*, *P. theae* [35], and *P. oenotherae* [36] have been reported to synthesize toxic substances. However, it remains unknown whether *P. colombiensis* possesses similar capabilities. This study did not investigate the pathogenic mechanisms of *P. colombiensis*; therefore, future research will focus on elucidating its pathogenicity in relation to *M. integrifolia*. Such investigations will provide a scientific basis for developing effective prevention and control strategies.

Several biological control agents have demonstrated efficacy in managing leaf blight caused by *Pestalotiopsis* spp. *Bacillus* spp. exhibit strong inhibitory effects against these pathogens [37,38]. Similarly, *Trichoderma harzianum*, *Dickeya* spp., and *Streptomyces* spp. have shown promising preventive and therapeutic potential [39,40,41]. Chemical fungicides such as Nerol and Flumax also display high efficacy in controlling *Pestalotiopsis* infections [42,43]. With increasing consumer demand for food safety and quality, along with stricter international trade standards for agricultural products, high-quality macadamia nuts have gained growing market popularity. Effective control of leaf blight is essential for maintaining healthy plant growth, improving fruit quality, and enhancing overall yield. Research into biological control strategies targeting pathogens responsible for macadamia diseases is emerging as a key trend in sustainable agriculture. Our team will continue to explore methods for preventing and managing *M. integrifolia* leaf disease caused by *P. colombiensis*, contributing to the refinement of theoretical frameworks in plant pathology while offering valuable insights for the management of other crop diseases.

*M. integrifolia* is widely consumed and highly valued globally as a nutritious and health-promoting food. In the early stages of macadamia cultivation in Yunnan, China, numerous Australian nut varieties were introduced and grown. The main production areas of macadamia nuts in Yunnan are concentrated in Lincang and Pu’er. Through adaptive breeding programs, several high-quality cultivars have been selected, including A4, A16, HAES660, and HAES788. Among these, seven varieties—A16, 816, Guang 11, HAES344, HAES660, Gui Re 1, and Changning 1—have been widely adopted in Yunnan [44]. The suitable *M. integrifolia* cultivars for Yunnan are comparable to those cultivated in Guangxi, Guizhou, the Hawaiian Islands, and Queensland, Australia. No cases of leaf blight have been reported in these regions, suggesting that the disease may become a significant constraint on *M. integrifolia* production, thus requiring further investigation. However, this study did not explore the relationship between cultivar type and disease incidence or assess the potential for disease-resistant varieties.

This research highlights that leaf blight could emerge as a major threat to macadamia production, underscoring the importance of developing effective disease management strategies. These findings provide a theoretical foundation for the precise prevention and control of *M. integrifolia* diseases, contributing to the optimization and localization of disease management practices. These efforts will support the healthy and sustainable development of the *M. integrifolia* industry in China. Furthermore, this study offers novel insights into the field of plant pathology and fills a critical research gap regarding pathogenic fungi affecting *M. integrifolia* in China, thereby holding significant academic value for future studies.

## Figures and Tables

**Table 1:** Primer sequences.

Locus	Primer	Sequence (5′–3′)	Tm (℃)	Reference
*TEFl-α*	EF1-728F	GTACCCGCTGAACTTAAGC	52	[20]
	EF2	GGA(G/A)GTACAGT(G/C)ATCATGTT		[21]
*LSU*	LROR	GTACCCGCTGAACTTAAGC	47	[22]
	LR5	ATCCTGAGGGAAACTTC		[23]
*ITS*	ITS1	TCCGTAGGTGAACCTGCGG	50	[24]
	ITS4	TCCTCCGCTTATTGATAT		

**Table 2:** GeneBank accession numbers used in the phylogenetic analyses.

Isolate	Country/Location	Host/Habitat	ITS	LSU	EF
*Pestalotiopsis telopeae*					
JX16b; CBS 113606; CBS 114161	China; Thailand	Unknown; *Telopea* sp.	OR781522	KM116202	KM199500
GZ22b; CBS 114137	China; Thailand	Unknown; *Protea* sp.	OR781519	KM116219	KM199559
*P. trachycarpicola*					
ZHKUCC 23-1026; AV-108; GUCC 23-0414	China; India	*Cave subculture*; *Persea americana*; Unknown	PP029403	PQ299650	PQ141020
PB2-e; AV-244; OP068	China; India; Thailand	*Panax notoginseng*; *Persea americana*; *Trachycarpus fortunei*	OR056327	PQ299673	JQ845946
YA3; AV-191; OP143	China; India; Thailand	Unknown; *Persea americana*; *Podocarpus macrophyllus*	OR484805	PQ299666	KC537816
*P. oryzae*					
CBS 171.26	Thailand	Unknown	KM199304	MH866374	KM199494
MWN35; Y2-3; CBS 111522	Kenya; China; Thailand	*Oryza sativa*; Unknown; *Telopea* sp.	OM899910	PQ432485	KM199493
*P. knightiae*					
KoRLI046217; NSW:7213; CBS 111963	Korea; Australia; Thailand	*Stereocaulon japonicum*; *Banksia serrata*; *Knightia* sp.	MN341554	OP082342	KM199406
CBS 114138	Thailand	*Knightia* sp.	KM199310	KM116227	KM199408
*P. biciliata*					
CBS 236.38	Netherlands; Thailand	Unknown; *Paeonia* sp.	MH855953	KM116214	KM199506
UMAS P16; CBS 124463	Malaysia; Thailand	*Shorea macrophylla; Platanus x hispanica*	KT337374	KM116224	KM199505
*P. parva*					
D034; CBS 265.37	Malaysia; Thailand	*Cocos nucifera*; *Delonix regia*	OP727543	KM116226	KM199508
CBS 278.35	Thailand	*Leucothoe fontanesiana*	KM199313	KM116205	KM199509
*P. humus*					
CBS 115450	Thailand	Unknown; *Ilex cinerea*	KM199319	KM116208	KM199487
CBS 336.97	Thailand	Unknown	KM199317	KM116230	KM199484
*P. australis*					
CBS 114193; CBS 114474; CBS 111503	Thailand	*Grevillea* sp.; *Protea susannae*	NR145239	KM116220	KM199557
CBS 114193	Thailand	*Grevillea* sp.	KM199332	KM116197	KM199475
*P. chamaeropis*					
CBS 186.71	Thailand; Netherlands	*Chamaerops humilis*; Unknown	KM199326	MH871839	KM199473
CBS 237.38; LC3619	Australia; Netherlands; China	*Prostanthera rotundifolia*; Unknown; *Camellia* sp.	KR259104	MH867450	KX895208
*P. macadamiae*					
BRIP 63739b; BRIP 70520	Australia; India	*Macadamia* sp.; *Pongamiea* sp.	KX186587	OP363152	MZ327136
BRIP 70519	Australia	*Macadamia* sp.	MZ303757	-	MZ327135
*P. colombiensis*					
CBS 118553	Thailand	*Eucalyptus grandis*	NR147551	NG069213	KM199488
5F; CBS 118553	Australia; Thailand	*Oryza australiensis*; *Eucalyptus grandis*	KU715149	KM116222	-
SWFUCB2	China	*Macadamia integrifolia*	PQ895603	PQ895623	PQ997934
SWFUCB1	China	*Macadamia integrifolia*	PQ895604	PQ895622	PQ997935
*Neopestalotiopsis saprophytica*					
CBS 115452	Thailand	*Litsea rotundifolia*	KM199345	KM116251	KM199538

“-” The database did not find this sequence.

## Data Availability

The original contributions presented in this study are included in the article. Further inquiries can be directed to the corresponding authors.
